# Complex Interaction between Resident Microbiota and Misfolded Proteins: Role in Neuroinflammation and Neurodegeneration

**DOI:** 10.3390/cells9112476

**Published:** 2020-11-13

**Authors:** Juliana González-Sanmiguel, Christina M. A. P. Schuh, Carola Muñoz-Montesino, Pamina Contreras-Kallens, Luis G. Aguayo, Sebastian Aguayo

**Affiliations:** 1 Department of Physiology, Universidad de Concepción, Concepción 4070386, Chile; julygonzalezsanmiguel@gmail.com (J.G.-S.); carmunozm@udec.cl (C.M.-M.); 2 Centro de Medicina Regenerativa, Facultad de Medicina Clínica Alemana, Universidad del Desarrollo, Santiago 7710162, Chile; cschuh@udd.cl (C.M.A.P.S.); paminaa.ck@gmail.com (P.C.-K.); 3 Program on Neuroscience, Psychiatry and Mental Health, Universidad de Concepción, Concepción 4070386, Chile; 4 School of Dentistry, Faculty of Medicine, Pontificia Universidad Católica de Chile, Santiago 8331150, Chile; 5 Institute for Biological and Medical Engineering, Schools of Engineering, Medicine and Biological Sciences, Pontificia Universidad Católica de Chile, Santiago 7820436, Chile

**Keywords:** Alzheimer’s disease, Parkinson’s disease, Creutzfeldt-Jakob disease, neuroinflammation, microbiome, periodontal diseases, biofilms, membrane permeability

## Abstract

Neurodegenerative diseases such as Alzheimer’s disease (AD), Parkinson’s disease (PD) and Creutzfeldt–Jakob disease (CJD) are brain conditions affecting millions of people worldwide. These diseases are associated with the presence of amyloid-β (Aβ), alpha synuclein (α-Syn) and prion protein (PrP) depositions in the brain, respectively, which lead to synaptic disconnection and subsequent progressive neuronal death. Although considerable progress has been made in elucidating the pathogenesis of these diseases, the specific mechanisms of their origins remain largely unknown. A body of research suggests a potential association between host microbiota, neuroinflammation and dementia, either directly due to bacterial brain invasion because of barrier leakage and production of toxins and inflammation, or indirectly by modulating the immune response. In the present review, we focus on the emerging topics of neuroinflammation and the association between components of the human microbiota and the deposition of Aβ, α-Syn and PrP in the brain. Special focus is given to gut and oral bacteria and biofilms and to the potential mechanisms associating microbiome dysbiosis and toxin production with neurodegeneration. The roles of neuroinflammation, protein misfolding and cellular mediators in membrane damage and increased permeability are also discussed.

## 1. The Burden of Neurodegenerative Diseases

Currently, nearly 50 million people worldwide suffer from neurodegenerative diseases (NDDs), mainly dementia, and this number is expected to reach 152 million by 2050 [1]. It is noteworthy that we are experiencing a shift in global demographics towards a large elderly population, which is increasing the prevalence of neurodegeneration worldwide and the financial burden associated with these diseases (e.g., medication, nursing care). For example, it is estimated that in the USA alone more than 5 million people aged 65 or older suffer from AD, and the costs of treating the disease are estimated at over US$180 billion per year [2,3].

In recent years, considerable progress has been made regarding the pathogenesis, diagnosis and treatment of Alzheimer’s disease (AD), Parkinson’s disease (PD) and Creutzfeldt-Jakob disease (CJD). However, these pathologies remain debilitating and fatal conditions, with significant negative medical, economic and social impacts. To date, there are no effective therapeutic approaches to prevent, delay or reverse these disorders, which start with cognitive loss and alterations of neurovegetative functions and progress towards language deficit, memory loss, motor difficulties and ultimately death [4]. These neurodegenerative diseases are associated with neuronal loss in several regions of the brain, such as the frontal cortex, hippocampus and basal ganglia. AD and PD can be classified as either “early-onset, genetic” (also known as “familial”) or “late-onset, sporadic” [5]. Most significantly, the late-onset forms are more prevalent and are considered to be the main cause of dementia and motor disease in the elderly population [6].

The most common neurodegenerative disease is AD, which is mainly characterized by marked cognitive dysfunction, impairment in the formation of new memories, and synaptic failure [7,8]. AD hallmarks include intracellular neurofibrillary tangles and the extracellular deposition of senile plaques that are mainly constituted by amyloid-β (Aβ) peptide [9]. Aβ is able to rupture the neuronal plasma membrane by the formation of pores leading to cytoplasmic leakage and cell death [10,11], by either direct lipid disruption or by its interaction with ion channels in the membrane [12,13]. Current research suggests that late-onset AD is mostly determined by environmental factors such as toxins, trauma and diet [14]. However, the underlying mechanism of action has not been completely elucidated yet.

The second most common neurodegenerative pathology is PD. In 2016, 6.1 million people worldwide were living with a diagnosis of PD, and it was estimated that 10 million people would be suffering from this disease by 2020 [15]. The prevalence of PD ranges from 100 to 200 cases per 100,000 people [16], and it affects nearly 3% of the population older than 65 years of age [17]. PD is mainly characterized by motor symptoms including bradykinesia, rigidity, tremor, postural instability, dysphagia and axial deformities, and non-motor symptoms such as cognitive dysfunction, sleep disorder, depression, anxiety, apathy, pain and dementia [17,18]. PD has been well described as the intraneuronal deposition of alpha synuclein (α-Syn), which contributes to the generation of protein inclusions known as Lewy bodies [19]. It is widely known that the loss of dopaminergic neurons in the substantia nigra pars compacta is the landmark physiopathological sign of the disease [18]. Due to the deleterious consequences of α-Syn, it has been considered a strategic target for future therapies to ameliorate the symptoms and slow down the progression of the disease.

Another relevant group of neurodegenerative disorders are prion diseases. There are three types of human prion disorders: sporadic, genetic and acquired [20]. The most common form of prion disease is sporadic CJD, a fatal pathology caused by misfolded prion proteins [20]. CJD is responsible for 85% of diagnosed prion disease cases, with a reported incidence of 1–2 cases per million people per year worldwide, and about 350 new annual cases in the United States [21]. The onset of CJD occurs in patients older than 67 years of age [20]. The main features of CJD and prion diseases are spongiform changes in gray matter, gliosis and neuronal death [22,23]. The most reported symptoms for CJD are progressive dementia, behavioral and cognitive impairment, insomnia, movement disorder and ataxia [24].

As a common feature, all neurodegenerative diseases seem to be associated with protein misfolding that leads to synaptic alterations, neuronal membrane damage and neuroinflammation. In addition, it has been recently suggested that microbial components, such as the ones present in the host microbiome, may also be actively involved in modulating neuroinflammation and protein misfolding. Therefore, in the present review we focus on the emerging hypothesis regarding the role of the host microbiome and its dysregulation in the onset of neurodegeneration, via (i) the entry of microbial cells, toxins and outer membrane vesicles directly into the brain, and (ii) the induction and maintenance of a systemic chronic inflammatory state. Furthermore, we discuss the involvement of associated systems such as the oral microbiome and bile, and potential routes of entry for bacteria and toxins into the central nervous system (CNS).

## 2. Protein Misfolding and Its Accumulation in Neurodegenerative Diseases

Neurodegenerative pathologies are commonly characterized by the misfolding, oligomerization and accumulation of toxic species such as Aβ in AD, α-Syn in PD, and the prion protein in CJD [24,25]. These protein alterations trigger neuronal degeneration and dysfunction and drive the progression of each particular disease [25]. For instance, there is abundant evidence demonstrating that Aβ peptide accumulation initiates and promotes AD. Aβ is mainly detected in the extracellular matrix in the brain and cerebrospinal fluid (CSF) at nanomolar concentrations, and is widely accepted as the main neurotoxic agent in the disease [26]. It is believed that early manifestations of AD are associated with the synaptotoxic effects produced by soluble oligomeric forms of Aβ [27]. The existence of mutations in genes for the amyloid-β precursor protein (AβPP) (chromosome 21) and presenilin 1 (chromosome 14) and 2 (chromosome 1) have also been reported in some AD patients, providing further evidence that Aβ is an important factor in the development of AD [28].

A well-accepted hypothesis for AD generation is that monomers of Aβ oligomerize, first forming low molecular weight species referred to as oligomers [27], which have been found to be highly neurotoxic to the membrane [13]. There is no clear consensus about the most toxic species, but there is an agreement that starting from dimers up to 56 kDa, oligomers are the most important causal agents in the disease [29,30]. These peptides/proteins can associate and damage the cell membrane, affecting neuronal function. The semipermeable property of the membrane is critical for cellular homeostasis, and the resulting Aβ-induced leakage of cellular components, as well as the non-regulated calcium influx into the cell, will turn into synaptotoxicity [13,31]. All the available evidence points to the idea that Aβ toxic events are multiple and that one/several of them might serve as a therapeutic target. Likewise, PD is mainly characterized by the formation of intracellular Lewy bodies in dopaminergic neurons. These structures are mostly formed by intracellular accumulation of α-Syn [24], a 140-residue protein encoded by the *Synuclein Alpha* (SNCA) gene that drives neurodegeneration. Prion diseases, on the other hand, have spongiform vacuolation, gliosis, neuronal loss and deposition of amyloid molecules immune-positive for prion protein (PrP) as hallmarks of the disease [24]. Thus, prion disorders are caused by the misfolded form of the prion protein, denoted prion protein scrapie (PrP^Sc^) [32]. The toxic misfolded PrP^Sc^ has a high content of β-sheet in its secondary structure, which generates a highly hydrophobic and insoluble protein with a high tendency to aggregate and form amyloid structures [24,32].

## 3. Protein-Induced Membrane Damage as a Central and Ubiquitous Player in Neurotoxicity

As discussed above, it is widely accepted that the accumulation of misfolded proteins is an important hallmark for AD, PD and CJD. Most importantly, these proteins are capable of inducing membrane damage in the brain by assembling monomers into non-selective ion pores and subsequently inserting them into a variety of cell membranes. For example, α-Syn oligomers increase the permeability of cell membranes in distinct types of neurons [33]. Additionally, α-Syn is also known to form pores in phospholipid bilayers found in mitochondria, inducing a complex series of multilevel conductance reminiscent of the effects of Aβ in hippocampal membranes [34]. α-Syn insertion into bilayers is facilitated by cardiolipin, an important phospholipid present in mitochondrial membranes [34]. As mitochondria are key organelles for cell energetic and ionic homeostasis, membrane alterations by oligomeric proteins can result in important alterations of cell viability.

Recent data using nanoelectrospray and mass spectrometry have shown that Aβ_42_ oligomerizes and forms β-barrel structure hexamers, which can be stabilized by the addition of lipids [35]. A similar situation is observed for toxic oligomers of PrP, associated with cellular membranes, where they might induce fast and prolonged toxic effects [36]. Studies in lipid bilayers, for example, have indicated that PrP oligomers cause a rapid and large increase in the permeability of the membrane, whereas monomeric forms cause no detectable leakage [36]. More recent studies using calcein-leakage assays showed that soluble prion oligomers are capable of producing leakage in negatively charged vesicles [37]. Studies at the nanometer level with atomic force microscopy showed that a fragment of the human PrP spanning residues 106–126 (PrP_106–126_) disrupted the intrachain conformation of phosphatidylcholine lipids [38]. All these results support the idea that, similar to Aβ and α-Syn, PrP oligomers can disrupt cell membranes. Further data regarding the relevance of these molecules in disease pathogenesis were obtained using the PrP_27–30_ fragment extracted from the brains of terminally ill golden Syrian hamsters infected with the 263K scrapie strain [39]. Interestingly, the electrophysiological recordings carried out with PrP resembled membrane responses obtained with Aβ in native neurons, including high variability on the amplitude of the unitary response and some spontaneous membrane breakages [11]. The responses showed a multistate conductance current, with at least one amplitude near 80 pS, a reversal around 0 mV and dependency on cation concentration (Na^+^ and K^+^). In addition, using the recombinant fragment of PrP (PrP_90–231_) a similar dependence on calcium was shown. In sum, AD, PD and prion diseases are associated with membrane alterations, increases in calcium permeability and ionic dyshomeostasis, which contribute to neurodegeneration. Most importantly, potentiation of local brain factors with other peripheral inflammatory mediators (such as those derived from a dysbiotic gut) may be associated with the progression of neurodegenerative diseases.

## 4. Neuroinflammation as a Common Factor across Neurodegenerative Diseases

As previously noted, synaptic and cellular alterations mediated by misfolded protein accumulation are the main hallmarks across NDDs [40]. However, all these diseases also share the common ground of displaying an increased inflammatory response in the brain, known as neuroinflammation. This process involves the activation of resident microglia and astrocytes that produce cytokines, chemokines and other inflammatory molecules within the CNS. Many of these markers are universal across NDDs, supporting the idea of a common neuroinflammatory profile across these diseases. Some of these common neuroinflammation mediators are chitotriosidase 1 (CHIT1), chitinase-3-like protein 1 (YKL-40), the glial fibrillary acidic protein (GFAP) and important pro-inflammatory cytokines, such as interleukin-1β (IL-1), IL-6 and tumor necrosis factor α (TNF-α) [41,42].

In general, for proteinopathies such as AD, PD and prion diseases, it has been shown that neuroinflammation can be directly induced by amyloids. In the context of AD, not only do reactive microglia colocalize with amyloid deposits in situ, but also in vitro Aβ oligomers have been shown to directly induce microglial activation [43,44,45,46,47]. Characterization of inflammatory molecules in CSF and plasma from AD patients has shown increased levels of pro-inflammatory cytokines such as IL-1β, IL-6 and TNF-α [42] and increases in the macrophage colony-stimulating factor, which has been described as a microglial activator [48]. Similarly, animal models of AD such as TgAPPsw and PSAPP transgenic mice also show an increase in a pro-inflammatory profile characterized by cytokines IL-1, IL-6 and TNF-α, and the granulocyte macrophage colony stimulating factor. This observation is consistent with *in vitro* studies using microglial cell cultures exposed to Aβ_42_ [48]. IL-12 and IL-23 were produced by microglia in AD transgenic mice models (APP/PS1), and the genetic ablation of these cytokines resulted in a decrease in cerebral amyloidosis [49].

In PD, microglia activation in the SNpc and striatum is well documented in murine models [50]. However, most of the microglial activation by α-Syn misfolding has been attributed to a deleterious pro-inflammatory response that is related to dopaminergic neuron degeneration [50,51]. As for AD, the cytokine profile in PD brains is characterized by the release of pro-inflammatory molecules IL-1β, IL-6, IL-12, interferon gamma (IFN-γ) and TNF-α [50,52]. Therefore, microglial response is an early marker of neuroinflammation in NDDs and seems to be the first mediator in the innate immune reaction in the CNS in these pathologies.

Studies in human prion diseases indicate that microglial activation correlates with the onset of the clinical signs and that its magnitude depends on prion strain [53,54]. Nevertheless, clustering analysis of neuroinflammatory gene expression performed in different brain regions of prion-infected mice suggested that astrocyte function is altered before microglia activation [55]. In this sense, transient prion neuroinflammation events show only partial similarity with the microglia degenerative phenotype reported in animal models of other NDDs, where microglial activation precedes astrogliosis. Regarding cytokine profiles in prion-induced neuroinflammation, similar markers to AD and PD such as TNF-α, IL-1β and particularly IL-1α are significantly increased in brain tissue from infected mice and CJD patients [56,57].

Since microglia are a key element in neuroinflammatory responses, and a predominantly inflammation-linked cytokine profile is found in AD, PD and prion diseases, microglial activation in these pathologies is considered to be associated with the pro-inflammatory M1 phenotype [58]. Nevertheless, anti-inflammatory cytokines such as IL-4, IL-10 and IL-13 are increased and have been detected in the striatum of PD patients [50]. Furthermore, increased levels of IL-4 and IL-10 have been found in CSF samples from AD [59] and CJD patients [60,61]. Due to recently developed one-cell transcriptome analyses, it has been possible to separately define specific phenotypic changes in microglia, astrocytes and neurons. In AD, these analyses have revealed microglial subpopulations with a distinctive molecular signature different from the classical M1 and M2 phenotype, which has led to the concept of disease-associated microglia (DAM) [62,63]. Two main receptors have been identified as key regulators in the generation of these particular phenotypes in neurodegenerative diseases: Toll-like receptors (TLRs) and triggering receptors expressed on myeloid cells-2 (TREM2) [62]. TREM2 interacts with two adaptor proteins, DAP12 and DAP10 [62]. Mutations in these proteins have been linked to AD, PD and other misfolding-related neurodegenerative disorders [64]. Even though the role of TREM2 signaling in neurodegeneration has not been defined, since both protective and harmful responses have been described, TREM2 has a clear role in the induction of the DAM phenotype [62]. For instance, in 5xFAD mice (a transgenic model of AD), single-cell transcriptome analyses revealed the existence of two DAM microglia clusters. Both clusters exhibited downregulation of homeostatic genes and upregulation of a particular signature that includes TREM2. In addition, TREM2 can act as a receptor for Aβ [65,66]. Similar microglial disease-specific phenotypes, distinguishable from the classic M1 phenotype induced by lipopolysaccharide (LPS), have been observed in other neurodegenerative disorders such as amyotrophic lateral sclerosis and multiple sclerosis [55,62]. Nevertheless, it is important to highlight that probably both elements, classical M1 and DAM, might be relevant in the progression of these diseases, with M1 contributing to the detrimental neuroinflammatory effects [62].

On the other hand, TLRs include 13 members that recognize different molecular patterns associated with pathogens, with LPS being one of the classical TLR inductors [62]. Besides pathogens, misfolded proteins may induce TLRs. In this sense, both α-Syn and Aβ have been described as TLR ligands [67,68]. Furthermore, some bacterial metabolites have also been described as ligands for TLR2 and TLR4 [69]. TREM2 has been also found to bind LPS, which is the most well-characterized bacterial-derived molecule in neurodegenerative disease models [70]. LPS is able to activate pro-inflammatory responses and contribute to detrimental effects in AD, PD and Huntington’s disease [71]. In the early stages of prion disease in ME7 prion strain-infected mice, LPS injection leads to exacerbated impairment in locomotor and cognitive functions [72]. Overall, LPS inoculation experiments suggest that bacteria-derived products could accelerate disease progression and contribute to neuronal decline.

Overall, activation of microglia is linked to the production of pro-inflammatory cytokines known to have deleterious effects when increased in tissues, including the brain [73]. IL-1β and TNF-α are able to reduce synaptic plasticity after acute application in brain slices [73]. Additionally, neurons express cytokine receptors that stimulate the mitogen-activated protein kinase (MAPK) family and lead to a reduction in synaptic efficiency [73]. Several calcium signaling mechanisms, including N-methyl-D-aspartate receptors (NMDARs), inositol trisphosphate receptor, ryanodine receptors and voltage-sensitive Ca^2+^ channels (VSCCs), may be modulated by cytokines in neurons. In this sense, increased levels of TNF-α can trigger calcium release from intracellular compartments and increase the expression of L-type VSCC. Neurons also express IL-1RAcPb, a neuron-specific IL-1 receptor accessory protein relevant for IL-1β binding that has been linked to an alternative phosphorylation pathway through Src phosphorylation, which is able to enhance Ca^2+^ influx through NMDAR activation [73].

In conclusion, AD, PD and prion diseases show early features of neuroinflammation that can be directly linked to misfolded protein deposition, which in turn triggers a specific microglia- and astrocyte-activated phenotype. However, external sources of neuroinflammation, different from those directly related to misfolded proteins, are also able to increase neuronal damage. In this sense, systemic inflammation could play an important role in the onset and maintenance of neuroinflammation; thus, the most recent evidence regarding the association between oral and gut microbiota and the promotion of an inflammatory state will be discussed, as well as its potential link with neurodegenerative diseases.

## 5. Human Microbiome Dysbiosis as a Source of a Systemic Chronic Inflammatory State

It is currently known that humans are inhabited by a wide and diverse range of microorganisms including bacteria, viruses and fungi, among others. These microorganisms, conjunctively known as the human microbiome, are compartmentalized in different areas of the human body such as the oral cavity, skin and gut; thus, each one of these “niches” holds a specific microbial composition. It is currently believed that we carry around more microbial cells on a daily basis than our own human cells [74]. Recently, it has been demonstrated that an overall healthy microbiome is crucial for maintaining homeostasis, and that imbalances in microbiota composition (i.e., dysbiosis) can lead to disease in many tissues and organs [75]. Systemic diseases such as cardiovascular disease, diabetes mellitus, rheumatoid arthritis and obesity are all believed to have a direct association with microbiome dysregulation, either via the direct effect of certain pathologic species or due to modulation of the host inflammatory response [76].

### 5.1. Human Biofilms: 3D Microbial Structures in Health and Disease

Most of our microbiome is not found in an unattached form, but instead as part of complex microbial communities known as biofilms. Biofilms are ubiquitous microbial structures found in most biological and non-biological environments [77]. In the human body, biofilms consist of surface-bound polymicrobial communities surrounded by an extracellular matrix that protects the biofilm from external injury such as mechanical forces or antibiotics [78,79]. Thus, biofilms are crucial for enhancing bacterial survival within the host. The formation of these biofilms is initiated by the attachment of bacteria onto surfaces, followed by bacterial division and the formation of a complex community. These biofilms are widespread throughout skin and mucosal surfaces within the mouth, gut, reproductive tract and urinary tract. Most importantly, a wide diversity of species within the biofilm is crucial for health, and dysregulation of residing species or imbalance in the number of organisms can lead to disease (known as biofilm-mediated infections or diseases) [80]. These biofilm imbalances are known to cause disease either by increasing the number of specific pathogenic species or by modulating the immune response towards chronic and/or destructive inflammation.

Oral biofilm-mediated diseases are good examples of the consequences of biofilm dysregulation within a specific niche. Dental caries, one of the most prevalent causes of dental pain and discomfort, is caused by a significant rise in the numbers of acid-producing species (such as *Streptococcus mutans* and lactobacilli) within the dental biofilm [81]. These acids are able to demineralize dental surfaces, which subsequently leads to cavitation and disease progression into deeper tissues within the tooth. One of the key factors behind the bacterial imbalance observed in dental caries is an increase in refined sugar consumption, and thus specific policies and strategies have been implemented worldwide in order to reduce sugar use in the population [81,82,83]. Furthermore, periodontal disease, a destructive inflammatory disease that affects the supporting tissues of teeth, is believed to arise from an imbalance of microbial species within the subgingival dental biofilm [84]. In periodontal disease, some specific pathogenic strains such as *Porphyromonas gingivalis* are able to increase their number within the dental biofilm and trigger a destructive inflammatory response by the release of proteases, enzymes and other bacterial components, as well as by modulating biofilm composition towards a dysbiotic state [84,85,86].

Interestingly, bacterial strains involved in oral and gut dysbiosis are known to play key roles in the development and progression of systemic diseases such as heart valvulopathies, diabetes mellitus, pre-eclampsia, rheumatoid arthritis and AD, among others [75,87,88,89,90]. Thus, local dysbiosis of oral and gut microbiota is known to not only impact local tissues but also affect distant organs, and there is mounting evidence that microbial elements may be associated with the development of neuroinflammation and neurodegeneration within the brain. Therefore, for the purposes of this review, we will focus on discussing recent evidence associating relevant oral and gut microbiota, as well as their dysbiosis, with AD, PD and prion disease.

### 5.2. Resident Oral Microorganisms and Their Association with AD and Neuroinflammation

Until recently, it was mostly believed that resident oral bacteria were only capable of generating disease confined within the oral cavity. However, current research has demonstrated that oral microbes are indeed associated with a wide range of systemic diseases and remote infections in other tissues and organs [88]. Although many oral species have been examined, for the purpose of this review we will focus on the most relevant organisms believed to be implicated with neurodegeneration.

#### 5.2.1. *Porphyromonas gingivalis*: Link between Periodontal Disease and Neurodegeneration?

One of the most prevalent oral diseases in adults and the elderly is periodontal disease. Although highly multifactorial, periodontal disease has an important bacterial component. Recent theories suggest that periodontal disease arises from dysregulation of the oral microbiome, which allows the overgrowth of highly virulent bacterial strains, paired with a destructive immune response from the host [91].

One of the most relevant bacteria in periodontal disease is *P. gingivalis*, a Gram-negative anaerobic bacterium that is part of the resident oral microbiome [92]. However, an increase in its proportion relative to other local microorganisms is associated with periodontal disease and tissue destruction [93,94,95]. Within periodontal disease pathogenesis, *P. gingivalis* is considered a “keystone” pathogen, as minor variations in its number within the biofilm can trigger enormous changes in the local environment [84]. Among others, *P. gingivalis* is known to modulate the host immune response in a biphasic manner: initially promoting inflammation to increase nutrient availability and biofilm growth, but subsequently facilitating bacterial resistance by destroying complement factors [96,97].

There is increasing evidence suggesting an important link between *P. gingivalis* and AD. Firstly, there is physical evidence of *P. gingivalis* components in brain samples of patients with AD. A recent study found *P. gingivalis* to be present in the brain of AD patients [98]. These authors also found gingipain, a toxic endopeptidase produced by *P. gingivalis*, to be present in AD brains and correlated with tau protein production. The inhibition of gingipain reduced infection of the brain and reduced neuroinflammation and Aβ_42_ production [98]. In another study, *P. gingivalis*-derived LPS was found in brain samples from AD patients [99]. These data are in line with previous work linking the presence of LPS from other Gram-negative bacteria, such as *Escherichia coli*, with increased Aβ deposition [100,101,102]. Recently, Haditsch et al. demonstrated that neurons derived from inducible pluripotent stem cells can be infected by *P. gingivalis* in vitro. Bacteria were found within the cytoplasm and lysosomes of affected neurons, which led to the formation of autophagic vacuoles, cytoskeleton disruption and loss of synapses [103]. Animal models have also demonstrated that *P. gingivalis* can migrate into the brain, as researchers demonstrated that ApoE^-/-^ mice infected with *P. gingivalis* were able to develop brain infections with the microorganism [104]. Another study by Ilievski et al. found that mice exposed to *P. gingivalis* developed neuroinflammation, neurodegeneration and extracellular deposition of Aβ [105].

Secondly, *P. gingivalis* may be linked with AD via its ability to modulate systemic inflammation. *P. gingivalis* (and other periodontal bacteria) is also known for promoting chronic inflammatory diseases such as diabetes, atherosclerosis and hypertension, which are also believed to be risk factors for the development of AD [106]. Kamer et al. found increased levels of TNF and antibodies against periodontal pathogens, including *P. gingivalis*, in AD patients compared to normal controls, suggesting an important link between periodontal bacteria and systemic inflammatory levels [107].

Furthermore, outer membrane vesicles (OMVs) may also play an important role in the development of AD. OMVs are 20–250 nm spherical buddings of the bacterial outer membrane containing lipids, proteins or nucleic acids [108,109]. For decades they were believed to be mostly a by-product of cell lysis; however, it is now known that their biogenesis is a deliberate and independent process [110]. Functions of OMVs have been associated with quorum sensing [111], as well as the distribution of virulence factors [112,113] and antibiotic resistance [114]. Within the gut microbiome, they have been shown to play a crucial role in gut homeostasis [115], carrying digestive enzymes [116] and modulating immune responses [117]. In the case of *P. gingivalis*, OMVs are important for immune response dysregulation and avoidance, tissue disruption and biofilm co-aggregation [118,119]. *P. gingivalis* OMVs are known to contain gingipain, LPS and other bacterial constituents [119,120,121], and previous research has demonstrated that OMVs are able to permeate the blood–brain barrier (BBB) [122] and thus could potentially be an important mechanism for entry into the CNS.

Finally, some recent data have also suggested the potential involvement of *P. gingivalis* in other NDDs such as PD. Adams et al. have demonstrated that RgpA protease produced by *P. gingivalis* is present in platelet-poor plasma clots from PD patient blood samples, and that *P. gingivalis*-derived LPS can induce hypercoagulability [123]. It is also believed that the systemic inflammatory state promoted by *P. gingivalis* and periodontal disease may also play an important role in PD pathogenesis [124]. However, further research is necessary to continue to unravel the association between this key bacteria and PD.

#### 5.2.2. Oral Spirochetes and Brain Infection

Another relevant group of microorganisms that has been associated with AD and brain infection is the spirochetes, and among these, dental spirochetes. Spirochetes are helical-shaped motile bacteria, with a remarkable ability to penetrate into tissues and disseminate infection [125]. Among these, *Treponema denticola* is regarded as an important periodontal pathogen, as its overgrowth is observed in periodontal disease sites and associated with tissue destruction. Spirochetes have been observed in the blood, CSF and brain tissue of AD patients [126], and recent investigations have identified numerous oral spirochetes as potential key players in brain infection in AD.

Riviere et al. found evidence for the presence of six oral *Treponema* species, namely, *T. amylovorum, T. denticola, T. maltophilum, T. medium, T. pectinovorum* and *T. socranskii*, in the frontal cortex of AD patients. Of 16 analyzed AD brains, 14 were positive for *Treponema*, versus only 4 out of 18 non-AD patients [127]. Authors also found evidence of oral *Treponema* within the trigeminal nerves and ganglion, and thus suggested that the microorganism is able to reach the brain directly via the peripheral nervous system instead of through the bloodstream. Furthermore, by employing a mouse model, Foschi et al. detected DNA from *T. denticola* in the brain and spleen of mice after dental pulp infection, further strengthening the idea that oral spirochetes can disseminate into the brain through both vascular and peripheral nerve routes [128].

The mechanisms behind the association between brain spirochetosis and AD remain debated. Spirochetes are believed to activate TLR on glial cells via CD14 and induce cytokine and pro-inflammatory molecule production, suggesting a potential mechanism of involvement in neuroinflammation and neurodegeneration [129,130]. Moreover, some authors suggest that some species of spirochetes are capable of synthetizing amyloidal-like fibrils [131], and previous research has suggested that Aβ itself may be produced in the brain as an antimicrobial peptide against invading pathogens [132]. A combination of bacterial-derived amyloid-like fibrils with local Aβ deposition and misfolding could potentiate neuroinflammation and potentially explain the association between spirochetes and neurodegeneration seen in some patients.

#### 5.2.3. Oral Fungi and Brain Infection and Inflammation

Another important component of the oral microbiome is fungi. Species such as *Candida albicans* are found ubiquitously on oral surfaces and are part of commensal biofilms. However, due to imbalances such as antibiotic usage or immunosuppressive conditions, they can overproliferate and cause local diseases such as oral candidiasis [133]. Interestingly, research has suggested that fungal infections can also migrate into the bloodstream and disseminate to distant tissues and organs. Recent studies have found the presence of fungal infection in the brains of AD patients [134]. Alonso et al. found evidence of fungal invasion in blood serum of AD patients, including *C. albicans* [135], and in a further study observed the presence of both fungal and microbial species in AD brain samples [136]. Similarly, Pisa et al. reported the presence of fungal material in the frontal cortex of AD patients, which was also found intracellularly [136], and further found fungal strains such as *Candida* spp., *Malasezzia* spp. and *Sacharomyces cerevisae* both intra and extracellularly in brain samples from AD patients [137]. Therefore, it is believed that the presence of *C. albicans* and other fungi inside the brain is associated with the development of AD [138]. There are many potential mechanisms explaining how fungal infection of the brain may promote AD. Most notoriously, Soscia et al. have shown that Aβ has an antimicrobial peptide behavior against *C. albicans* [132]; thus, Aβ deposition may be part of a neuroinflammatory response to clear the fungi from the brain. It is also known that disseminated fungal invasion can increase systemic cytokine production and activate both innate and adaptative immunity [139], which could potentiate neuroinflammation in the brain. Furthermore, some fungi such as *Candida* have the ability to secrete amyloid-like substances that may serve a similar function to Aβ inside the brain [140]; however, the effect of these fungal amyloid-like molecules on neuronal viability remains to be explored.

Interestingly, a recent study by Wu et al. employed a mouse model to generate *C. albicans* intravenous infections and observed the development of neuroinflammation, and accumulation of activated glia cells and Aβ around yeast cells. Within the brain, activation of transcription factor NF-κB and increases in IL-1β, IL-6 and TNF-α were also observed as a result of *C. albicans* invasion, which activated the local innate immune response. As a result of this neuroinflammation, infected mice showed mild memory impairment associated with *Candida* infection, which cleared after antifungal treatment [141]. Overall, fungal invasion of the brain appears to induce local neuroinflammation via similar molecules to the ones traditionally described in NDDs, and thus may potentiate neurodegeneration in some patients.

### 5.3. Resident Gut Bacteria and Their Association with Neuroinflammation and Neurodegeneration

**Figure 1:**
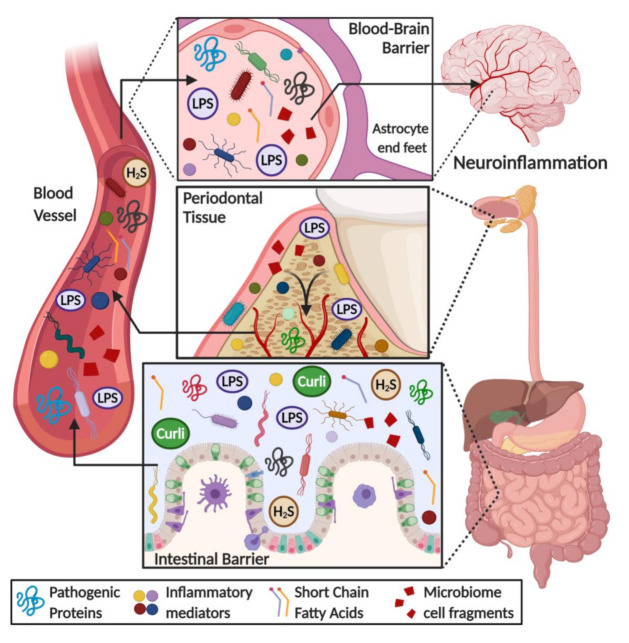
Overview of the role of gut and oral microbiome in the onset of neuroinflammation in neurodegenerative diseases. Some microbial products, such as lipopolysaccharide (LPS), short chain fatty acids, hydrogen sulfide (H_2_S), amyloid-like substances (i.e., curli protein), bacteria cell fragments and pro-inflammatory mediators (i.e., cytokines, chemokines, ROS species), are released into the bloodstream because of an increase in gut-blood barrier permeability. These metabolites flow through the circulatory system reaching the brain, where they can permeate a weakened blood–brain barrier, triggering a neuroinflammatory response and worsening the pathological hallmarks of neurodegenerative diseases.

Similar to the oral microbiota, the gut is home to trillions of resident microorganisms that are essential for our health and well-being, and that are able to influence health and disease locally and systemically. The resident gut microbiota participates in numerous important processes such as nutrient digestion and local gene expression and immune system regulation [142]. Most importantly, alterations in gut microbiota composition are associated with the onset and progression of many chronic inflammatory diseases in humans (reviewed by [143] and [90]). Among these remote effects, it has been shown that there is an intimate bidirectional connection between the gut and brain, known as the “gut-brain axis”, which is believed to regulate behavior, anxiety and pain [144,145]. There are currently many potential explanations associating alterations in the gut microbiome with NDDs, including the passage of microbial cells and products into the brain as well as potentiation of neuroinflammation via inflammatory mediator production (Figure 1).

#### 5.3.1. Gut Microbiota Dysbiosis Generates a Pro-Inflammatory State

Observational studies in recent years have suggested an association between gut microbiota alterations and AD. Vogt et al. observed that AD patients have reduced gut microbial diversity compared to controls, as well as compositional changes such as decreased Firmicutes and increased Bacteroidetes compared to control patients [146]. In a recent study, Sanguinetti et al. showed that mice in a pre-dementia state have reduced microbial gut diversity and altered bacterial proportions compared to control mice [147]. Another study by Minter et al. utilizing the APP_SWE_/PS1_DE9_ AD mouse model demonstrated that shifting gut microbiome composition with antibiotic treatment decreased Aβ plaque deposition and alterations in cytokine and chemokine levels in circulation, such as the increase in CCL11 believed by the authors to lead to Aβ phagocytosis in the brain [148]. Interestingly, germ-free APP transgenic mice show a significant reduction of Aβ pathology in the brain, strengthening the notion of microbial involvement in AD pathogenesis [149].

There is also mounting evidence of a correlation between gut microbiota and PD. Forsyth et al. found an association between increased gut leakiness and the presence of PD, which was also accompanied by an increase in *E. coli* and α-Syn in the intestine [150]. The authors suggested that this local increase in α-Syn may be a consequence of the pro-inflammatory state in the region generated by microbial components such as LPS. Research by the same group found significant differences in the composition of fecal microbiota between PD and healthy patients, as PD patients had decreased amounts of Firmicutes compared to controls [151], similar to what is observed in AD patients [146]. In the same study, authors also noted that PD duration was positively correlated with Bacteroidetes and negatively correlated with Firmicutes. Interestingly, they also observed that genes involved in pathways such as LPS biosynthesis and bacterial secretion were increased in PD patients compared to controls. Recently, Sampson et al. observed that bacteria that produce curli, a bacterial aggregating amyloid, were found to promote α-Syn pathology in both the gut and brain and potentiate motor abnormalities in a mouse model [152]. Although there are still doubts as to how intestinal α-Syn and amyloid formation may impact the brain in PD, some research has suggested the possibility that α-Syn may spread via the vagus nerve to the brainstem [153].

Furthermore, in a recent clinical study, Cattaneo et al. found that cognitively impaired patients with brain amyloidosis expressed a decreased abundance of the anti-inflammatory *Eubacterium rectale* and a higher abundance of inflammatory strains such as *Escherichia* and *Shigella* compared to healthy controls and amyloid-free cognitively impaired patients [154]. These microbiota alterations were associated with increased levels of pro-inflammatory cytokines such as IL-6, NLRP3, CXCL2 and IL-1β in the amyloid-positive group and correlated with the overabundance of *Escherichia/Shigella*. Overall, it seems that the gut microbiota composition is crucial in maintaining inflammatory homeostasis, and alterations of diversity or relative proportions between species can trigger or maintain chronic inflammatory states by the modulation of pro-inflammatory cytokine production, among others. Further strengthening this hypothesis is the fact that probiotic treatments that regulate imbalances in the gut microbiota have shown an important protective effect against inflammation, cognitive decline and AD development [155,156,157,158,159,160]. Administration of probiotic strains such as *Lactobacillus plantarum* P8 was recently shown to improve cognition, learning and memory in a group of stressed adults [161].

Regarding potential mechanisms behind the neuroprotective effect of probiotic administration, Bonfili et al. observed that a probiotic formulation of lactic acid and bifidobacteria was able to potentiate the proliferation of anti-inflammatory species, which in turn modulated gut hormones and peptides that reduced Aβ load and improved cognitive function [162]. Authors believe that this effect was mediated by the SIRT1 pathway, a strong neuroprotective and antioxidant molecule in the brain of treated mice that reduces Aβ and tau accumulation. Furthermore, Wang et al. found that the combined administration of *Bifidobacterium bifidum* TMC3115 and *Lactobacillus plantarum* 45 improved spatial memory in an AD mouse model, and was associated with the regulation of gut homeostasis via an increase in microbiota diversity and a reduction of the abundance of Bacteroides species [163].

#### 5.3.2. *Helicobacter pylori*: A Crucial Species for Chronic Inflammation and AD

Within the gut microbiome, one microbe believed to be a key player in chronic inflammation is *Helicobacter pylori*. For years, it has been known that *H. pylori* is a causative agent of local pathologies such as stomach ulcer and gastric cancer, mainly due to protease and cytotoxin production [164], as well as local immune modulation via TNF-α and IL-1β [92,93,94,95]. Recent clinical studies have observed a correlation between *H. pylori* infection and many chronic inflammatory diseases including AD [165,166]. Furthermore, *H. pylori* eradication has been associated with reduced progression of dementia [167,168]. Shen et al. found that APP/PS1 mice expressing AD had an increased abundance of *Helicobacter* within their gut microbiota compared to healthy mice [169].

Similar to the effect of other microorganisms, the mechanisms behind the link between *H. pylori* and AD seem to be multifactorial but mostly mediated by a sustained chronic inflammatory response with systemic effects. *H. pylori* infection increases the production of pro-inflammatory mediators such as TNF-α, IFN-γ and interleukins that are believed to be important in neuroinflammation [170,171]. Some reports have found an increase in IL-8 and TNF-α in the CSF in *H. pylori*-infected patients [172]. Furthermore, a *H. pylori*-derived peptide known as Hp(2-20) was found to alter the expression of 77 AD genes, many of which are known to modulate inflammatory pathways [173].

Questions remain as to whether *H. pylori* can effectively invade and infect the CNS and trigger AD by direct brain colonization [166]. However, a possible mechanism might be found within the known interplay of *H. pylori* and its OMVs in modulating cell–cell contacts on several levels. Secretion of serin protease HtrA leads to the cleavage of occludin and claudin-8 (tight junctions) and E-cadherin (adherens junction). Furthermore, virulence factor CagA (cytotoxin-associated gene A) acts on apical-junctional complexes, activates β-catenin and, in its phosphorylated form, can induce cell scattering and morphological changes (reviewed by [174]). In addition, *H. pylori* OMVs have been found to carry CagA and to strongly associate with tight junctions, adding another route of modulation [175]. Although the mechanisms mentioned above have mostly been explored in the context of gastric cancer, this gut barrier destruction could promote the migration of microorganisms into other tissues, such as the brain. Nevertheless, the importance of *H. pylori* in neurodegeneration is not fully known, and future work is needed to explore the potential mechanistic explanations behind this association.

#### 5.3.3. *Akkermansia muciniphila*: An Important Regulator of Inflammation in the Gut

*Akkermansia muciniphila* appears to be one of the key regulators of inflammation in the gut. *A. muciniphila* is part of the phylum Verrucomicrobia, a relatively understudied phylum due to its difficult cultivation in laboratory conditions. In an attempt to associate microbial involvement with the development of Aβ pathology, Harach et al. found that a decrease in *A. muciniphila* was correlated with the progression of Aβ in the brain [149]. These findings were confirmed by Ou et al. who found that increasing *A. muciniphila* resulted in a reduction in Aβ_40_ and Aβ_42_ levels in the cerebral cortex of AD model mice (APP/PS1), and improved learning and completion rates in maze tests [176]. A possible pathway could be via the involvement of TLR4 in AD as aggregated Aβ can bind TLR4 and subsequently activate microglia, resulting in increased cytokine production (reviewed by [177]). Furthermore, several studies by Ashrafian et al. demonstrated that *A. muciniphila* OMVs have the ability to decrease TLR4, resulting in decreased inflammation [178,179]. Moreover, the absence of *A. municiphila* has also been noted recently in other inflammatory diseases such as autistic disorders [180,181] and depression [182].

Interestingly, the abundance of *A. muciniphila* has been shown to have the reverse correlation in PD. Several studies found an increase in fecal *A. muciniphila* with the progression of symptoms (reviewed by [183]). To date, the discrepant effect of *A. muciniphila* in AD and PD has not been discussed. However, one potential explanation is that alterations in *A. municiphila* abundance may actually be a consequence of neurodegeneration, as one of the main symptoms in PD is a reduction in gut motility due to the involvement of the vagus nerve and the enteric nervous system. Supporting this idea is the fact that several studies in chronic constipation patients reported a microbiota profile similar to the one in PD: an increased abundance of *A. municiphila* together with a decrease in *Prevotella* [184,185,186,187,188,189]. Nevertheless, further research is needed to determine the exact association between this bacterial strain and neurodegenerative diseases.

#### 5.3.4. Bile Acids and Their Potential Role in Neurodegenerative Diseases

The role of bile acids in inflammation has become an emerging topic in recent years. Synthesized by the liver, bile acids (BAs) are stored in the gallbladder, released into the small intestine, and play a key role in emulsifying dietary fats as well as in the absorption of lipids and lipophilic vitamins. Overall, BAs can be divided into primary BAs and secondary BAs. While primary BAs are produced by the liver, the gut microbiome modulates and metabolizes these primary BAs into secondary BAs [190]. Hence, the gut microbiome and BAs are strongly interconnected. One the one hand, BAs act against overgrowth of specific bacteria (e.g., lactobacilli or bifidobacteria [191]), and on the other hand, the microbiome has been shown to affect BA composition and metabolism in the liver (reviewed by [192]). Previous sections have described how gut dysbiosis itself can alter neuroinflammation, which can be extended to BAs and their antibacterial effect on known inflammation-regulating bacteria such as bifidobacteria. However, serum BAs also appear to play physiological roles in the brain, displaying a neuroactive potential in several neurotransmitter receptors in the brain such as γ-aminobutyric acid type A (GABA_A_) receptor and NMDARs [193]. Furthermore, they also act as agonists for the G-protein coupled bile acid receptor 1 (Gpbar1 or TGR5), mediating cyclic adenosine monophosphate (cAMP) signaling [194], and have been shown to be ligands for farnesoid X receptor (FXR), a nuclear transcription factor [195].

The BA receptor FXR has been associated with a number of AD-related mechanisms. FXR overexpression appears to play a role in Aβ-triggered neuronal apoptosis. It has been speculated that interaction with the cAMP-response element-binding protein (CREB) leads to its decrease, as well as a decrease in brain-derived neurotrophic factor (BDNF) protein levels [196]. Furthermore, Vavassori et al. demonstrated that FXR in the gut is associated with intestinal immunity. Activation of FXR by LPS-activated macrophages results in a downregulation of NF-κB-dependent genes IL-1β, IL-2, IL-6, TNF-α and IFN-γ [197].

Several BAs have been associated with neurodegenerative diseases (e.g., deoxycholic acid DCA), and among these is tauroursodeoxycholic acid (TUDCA), a secondary bile acid that has displayed a neuroprotective effect in PD [198], AD [199,200] and prion disease models [201]. Interestingly, TUDCA appears to act on several levels. In PD, TUDCA was shown to decrease degeneration of dopaminergic neurons caused by 1-methyl-4-phenyl-1,2,3,6-tetrahydropyridine (MPTP) [198]. Furthermore, as PD has been associated with impaired mitochondrial function and an increase in oxidative stress, Rosa et al. found a TUDCA-associated upregulation of the mitochondrial turnover [202]. In AD, Nunez et al. demonstrated that TUDCA reduced amyloid plaques in the frontal cortex and hippocampus, and improved memory retention [203]. Wu et al. assessed the effect of TUDCA in LPS-induced cognitive impairment and discovered that TUDCA reverses LPS-induced TGR5 downregulation, and therefore prevents hippocampal neuroinflammation by NF-κB signaling [204]. In prion diseases, TUDCA has been found to act on yet another mechanism, namely by blocking or interfering with the conversion of prion protein (PrP^c^) into its misfolded form PrP^Sc^, therefore reducing neuronal loss [201].

**Figure 2:**
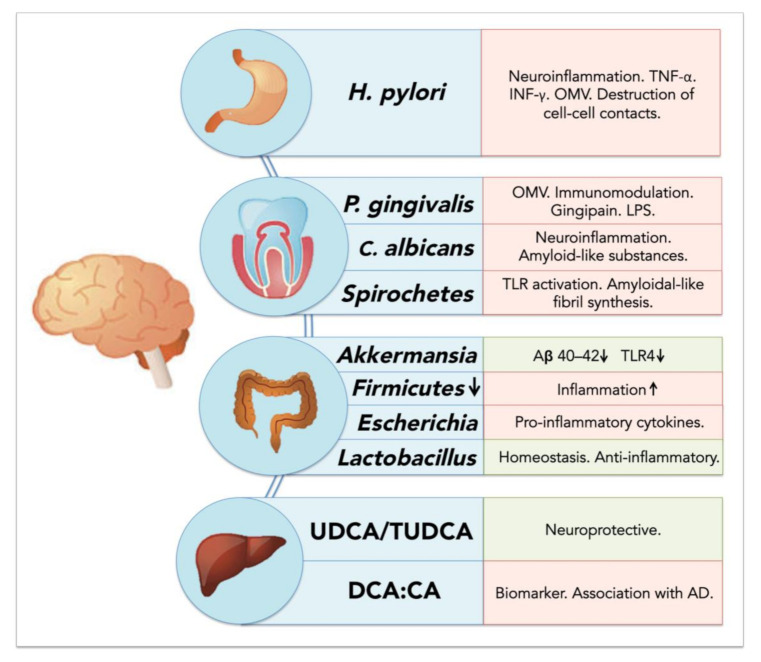
Schematic overview of microbiome-associated factors influencing neurodegenerative diseases. Factors are displayed according to their organ of origin (stomach, oral cavity, colon and liver); positive effects on neurodegenerative diseases are displayed in light green, negative effects in light red.

The ratios between different BAs appear to be important in the development of neurodegenerative diseases (Figure 2). For example, an increased ratio of the secondary BA deoxycholic acid compared to the primary BA cholic acid has been associated with cognitive decline [205]. Interestingly, Firmicutes such as Clostridiaceae, Lachnospiraceae and Ruminococcaceae, responsible for 7α-dehydroxylation of cholic acid (CA), have been found to be significantly decreased in AD [206,207], and the association between the increased DCA:CA ratio has not yet been elucidated. However, increased levels of DCA have been associated with increased permeability of the BBB through phosphorylation of occludin [208], and thus may also play an important role in disrupting barriers and facilitating the entry of other microorganisms into the brain.

## 6. Direct and Indirect Effects of Microbiota on the Brain: Role of Barrier Evasion and Permeability

The CNS is one of the tissues that benefits from a degree of antigen tolerance, also known as immune privilege. This characteristic is mainly due to the presence of the blood–brain barrier (BBB), which separates the CNS from the systemic immune response and protects the brain and spinal cord from acute inflammatory mediators, which could induce more damage than immune control. This control is not only exerted by the BBB but also by the blood-cerebrospinal fluid barrier (BCSB) and the arachnoid barrier [40]. These barriers also explain why antigen emergence within the brain or spinal cord does not generate a peripheral immune response. The BBB is a complex and highly regulated exchange interface, composed of pericytes, astrocytic processes and nearby neurons adjacent to capillaries. It works as a carrier, an enzymatic barrier, a paracellular barrier (due to endothelial junctions) and a cerebral endothelium [209,210]. During systemic inflammation, both disruptive and non-disruptive changes in the BBB can be observed. Although no visible changes are produced with non-disruptive BBB damage, the changes in BBB physiology might alter astrocyte function and cytokine production, and higher levels of pathogen invasion can be produced [210]. Moreover, under non-disruptive alterations, very few molecules can cross the barrier. On the other hand, during disruptive events such as those induced by bacteria-derived LPS, histological and anatomical changes can be observed with strong alterations in permeability. In several neurodegenerative diseases such as AD, the BBB is also affected and its role in CNS permeability is compromised. In the case of AD, abnormal clearance of Aβ and an increased BBB permeability allowing the entrance of pro-inflammatory molecules into the brain are observed [210]. In a global systematic context, three membranous locations are critically important because of their physicochemical properties: membranes of the gastrointestinal–blood barrier, of the BBB, and finally the semipermeable neuronal membrane. It has been proposed that because of an increased leakage in both the gastrointestinal–blood barrier and the BBB in AD (and perhaps other NDDs), these pathologies might be considered as “defective barrier” diseases [69,211]. This increased permeability would facilitate the entry of bacterial cells, bacterial molecules and peripheral inflammatory mediators into the brain that subsequently would exacerbate local neuroinflammation via the mechanisms mentioned previously.

## 7. Microbiome Dysbiosis and Neuroinflammation: A Complex “Toxic” Mixture Affecting the Brain during NDD

**Figure 3:**
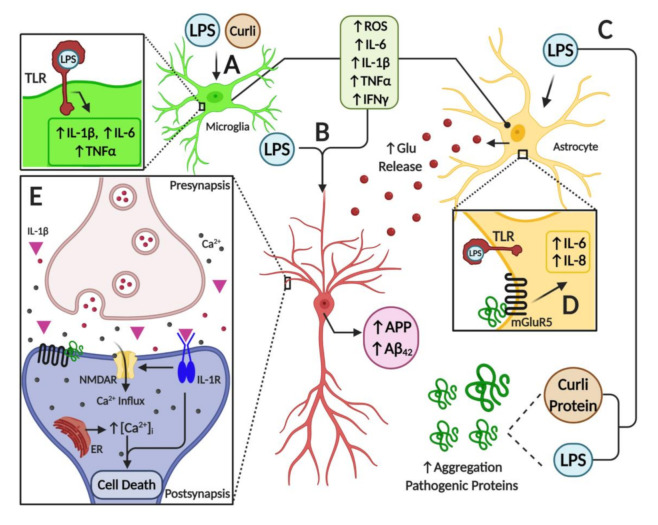
Illustration of neuroinflammatory mechanisms mediated by microbiome-derived products in nervous tissue. (**A**) Toll-like receptors (TLRs) expressed in glial cells are activated by LPS, triggering the activation of astrocytes and microglial cells. This activation induces an inflammatory response by overexpression and release of pro-inflammatory cytokines such as IL-6, IL-1β, TNF-α and IFN-γ, and by an increase in oxidative stress due to the generation of reactive oxygen species. Furthermore, bacterial amyloid proteins (curli) activate glial cells and induce the expression of pro-inflammatory mediators. (**B**) Pro-inflammatory mediators, together with LPS, increase the expression of the amyloid precursor protein (APP), and the deposition and misfolding of Aβ peptide. (**C**) Both LPS and curli are able to increase the deposition and aggregation of pathogenic proteins. (**D**) In astrocytes, among other cell types, activation of mGlurR5 receptor by pathogenic proteins triggers the overexpression of pro-inflammatory cytokines such as IL-6 and IL-8, which worsen the inflammatory milieu in the brain. Moreover, a high level of pro-inflammatory mediators leads to increased levels of the neurotransmitter glutamate, furthering ionic dyshomeostasis and augmenting neuronal excitotoxicity. (**E**) Finally, mGluR5 activation by pathogenic proteins induces the release of calcium from the endoplasmic reticulum, leading to ionic and mitochondrial dyshomeostasis, which results in neuron death. Furthermore, the activation of IL-1R in neurons by the binding of IL-1β cytokine amplifies the activity of NMDARs and mediates the inflammatory response via p38 MAPK. Overall, these alterations stimulate endoplasmic reticulum (ER) Ca^2+^ release through ryanodine receptors and IP3 receptors, which trigger ER stress and mitochondrial fragmentation leading to synaptic failure and neuronal apoptosis.

As discussed throughout this review, it appears that a major source of pro-inflammatory diffusible signals associated with brain neuroinflammation originates from peripheral organs and systems such as the gastrointestinal (GI) tract microbiome. Bacterial components such as LPS, which can enter the bloodstream, stimulate systemic pro-inflammatory responses in the host including the CNS. At the cellular and molecular levels, LPS is able to induce the release of inflammatory mediators and eventually induce synaptic loss, which can lead to cognitive impairment via microglial activation, generation of reactive oxygen species (ROS) and oxidative stress [69] (Figure 3). It has been proposed that bacterial LPS may be involved in neuroinflammation associated with amyloid fibril formation in AD [212], suggesting that LPS acts as a promoter of Aβ fibrillogenesis in a time-dependent manner, possibly through a heterogeneous nucleation mechanism. It has also been shown that a single intraperitoneal injection of LPS increases Aβ_42_ levels and astrocyte activation in critical brain regions such as the cerebral cortex and hippocampus [101]. In addition, LPS affected memory in mice, suggesting the development of brain dysfunction [101]. These negative actions of peripheral LPS on amyloidogenesis, memory function and neuronal death were inhibited by sulindac, an anti-inflammatory agent, supporting the role of peripheral inflammation in AD pathology. Interestingly, it was shown that inflammatory cytokines such as IL-1β and TNF-α can increase the expression of APP and the formation of Aβ [213,214].

Furthermore, several studies have shown the interplay between toxins released by bacteria and neurodegeneration. For example, some Enterobacteria species may release amyloid peptides that alter the aggregation of α-Syn in the brain [215]. Another study also showed that when aged rats were exposed to curli-producing *E. coli*, an increased neuronal α-Syn deposition in both the gut and brain was observed; furthermore, animals also showed enhanced microgliosis and astrogliosis compared to those exposed to control bacteria unable to synthesize curli [216]. Rats exposed to curli also showed a higher expression of TLR2, IL-6 and TNF-α in the brain [216]. Overall, it appears that signals released by bacteria can modulate amyloid formation and activate pro-inflammatory responses in the brain, suggesting a strong interplay between the microbiome and neuroinflammation in neurodegenerative diseases (Figure 3).

The potential link between bacterial-derived products and neurodegeneration is strengthened by several other studies. For example, a reduction of several Aβ species in the brain and blood was detected in APPPS1 transgenic mice in the absence of gut microbiota [149]. Therefore, the presence of a GI germ-free condition reduced cerebral Aβ amyloid pathology in diseased mice when compared to control mice with control intestinal microbiota. Furthermore, the colonization of germ-free APP transgenic mice for 8 weeks with microbiota from conventionally raised APP transgenic mice increased Aβ_42_ levels [149]. Overall, these results support the idea that the GI microbiota is involved in the development of Aβ pathology in the brain, as well as the existence of pro-inflammatory mediators with the ability to enter the CNS and produce a local response. On the other hand, short-chain fatty acids derived from the GI microbiota can inhibit amyloid aggregation [217]. Additionally, it seems feasible that GI-derived amyloid and toxins might activate signaling pathways affecting neuroinflammation and the pathogenesis of AD [218].

Furthermore, the association between misfolded proteins and cellular membrane damage is also modulated by the activation of membrane receptors that influence the neuroinflammatory response in the brain. For instance, the enhancement of inflammatory markers released from brain astrocytes is associated with AD and PD [219]. Additionally, it is believed that metabotropic glutamate receptor 5 (mGluR5) exerts an important action on neuroinflammation, affecting cytokine expression and activation of glial cells, such as microglia and astrocytes in the brain [219,220] (Figure 3). Activation of mGluR5 results in the stimulation of phospholipase C and phosphoinositide hydrolysis, leading to intracellular Ca^2+^ mobilization and activation of extracellular signal-regulated kinases 1 and 2 (ERK1/2) downstream signaling pathways, which might further affect neuroinflammation. mGluR5 activation contributes to a dysregulated rise in intracellular calcium concentration that is deleterious for neurons in AD and PD. For example, the exposure of neurons to Aβ oligomers induces mGluR5-dependent release of Ca^2+^ from the endoplasmic reticulum and toxicity [221,222]. This was corroborated using an mGluR5 knockout (KO), which showed reduced neutrophil infiltration and inflammatory cytokine expression in the brain at 24 h post-insult accompanied by improved neurological function [223]. In addition, mGluR5 KO showed reduced damage to BBB integrity and permeability, which might affect the influx of inflammatory modulators and peripheral cells into the brain. Interestingly, activation of these metabotropic receptors led to increases in intracellular calcium, further potentiating its increase due to direct membrane damage by these oligomeric toxic complexes.

## 8. Conclusions

Alzheimer’s disease, Parkinson’s disease and prion disorders are debilitating brain diseases affecting millions of people worldwide. The presence of misfolded proteins such as Aβ, α-Syn and PrP^Sc^ depositions in the brain is a common feature in these conditions, leading to synaptic disconnection and subsequent progressive neuronal death. In addition, extensive recent work suggests an association between host microbiota, neuroinflammation, neurodegeneration and dementia. The present review points toward the idea that these diseases are comprised of a mixture of endogenous and exogenous altered proteins and diffusible inflammatory mediators that act synergistically to cause neurodegeneration and dementia. The toxic determinants seem to be potentiated by bacterial brain invasion following barrier leakage, and the release of toxin and inflammatory products due to changes in the immune response. The release of cytokines and LPS, together with the accumulation of misfolded proteins (Aβ, α-syn and PrP^Sc^) acting as membrane pores and the activation of ionotropic and metabotropic receptors, all lead to an increase in intracellular calcium and subsequent ionic dyshomeostasis, leading to toxic exacerbation. Therefore, controlling these cellular and microbial determinants might prove helpful for the prevention and future treatment of neurodegenerative diseases.
